# Causal inference with large‑scale assessments in education from a Bayesian perspective: a review and synthesis

**DOI:** 10.1186/s40536-016-0022-6

**Published:** 2016-05-03

**Authors:** David Kaplan

**Affiliations:** Department of Educational Psychology, Universityof Wisconsin-Madison, Madison, Wisconsin, USA

## Abstract

This paper reviews recent research on causal inference with large-scale assessments in education from a Bayesian perspective. I begin by adopting the potential outcomes model of Rubin (J Educ Psychol 66:688–701, 1974) as a framework for causal inference that I argue is appropriate with large-scale educational assessments. I then discuss the elements of Bayesian inference arguing that methods and models of causal inference can benefit from the Bayesian approach to quantifying uncertainty. Next I outline one method of causal inference that I believe is fruitful for addressing causal questions with large-scale educational assessments within the potential outcomes framework— namely, propensity score analysis. I then discuss the quantification of uncertainty in propensity score analysis through a Bayesian approach. Next, I discuss a series of necessary conditions for addressing causal questions with large-scale educational assessments. The paper closes with a discussion of the implications for the design of large-scale educational assessments when the goal is in asking causal questions and warranting causal claims.

## Background

With the reauthorization of the United States Elementary and Secondary Education Act (referred to in 2001 as *No Child Left Behind–NCLB*), attention focused on the need for evidenced-based education research, particularly education policies and interventions that rest on what NCLB referred to as “scientifically based research.” In practice, this focus on scientifically based education research translated into a preference for research studies based on the principles of randomized experimental designs. Specifically, Part A., Sec. 9101 of the No Child Left Behind Act, under the definition ‘Scientifically Based Research” stated

“The term ‘scientifically based research’(A) means research that involves the application of rigorous, systematic, and objective procedures to obtain reliable and valid knowledge relevant to education activities and programs; and (B) includes research that ... (iv) is evaluated using experimental or quasi-experimental designs in which individuals, entities, programs, or activities are assigned to different conditions and with appropriate controls to evaluate the effects of the condition of interest, with a preference for random-assignment experiments, or other designs to the extent that those designs contain within-condition or across-condition controls;...”

Although randomized experimental and quasi-experimental designs can, under ideal conditions, provide a sound basis for evaluating causal claims, this does not preclude the possibility that reliable causal inferences can be drawn from non-experimental/observational settings.

For our purposes, the goal is to address causal questions in the context of large-scale educational assessments (LSAs). Examples of such LSAs include national surveys such as the Early Childhood Longitudinal Study (ECLS-K) and the National Assessment of Educational Progress (NAEP) in the United States, but also cross-national surveys such as the Organization for Economic Cooperation and Deveopment (OECD)’s Program for International Student Assessment (PISA) and the International Association for the Evaluation of Educational Achievement (IEA)’s Program on International Reading Literacy Study (PIRLS). There is an increasing desire among policymakers charged with administering LSAs to begin to address questions from a causal inferential framework and so new thinking about the problem of causal inference with LSAs is required.

The purpose of this paper is to review and synthesize recent work by the author on the issue of causal inference with LSAs. In considering causal inference in any empirical setting, a theory of causal inference is needed in which to situate causal questions. Theories of causality abound (see Cartwright 2007); however for this paper, I situate causal inference with LSAs in the context of the *potential outcomes* framework of Rubin (1974). The statistical model that I focus on that arguably best illustrates the utility of the potential outcomes framework for LSAs is *propensity score analysis* (Rosenbaum and Rubin 1983). In addition, I examine propensity score analysis from a Bayesian perspective primarily because the Bayesian framework explicitly allows the analyst to incorporate what is reasonable to believe about the causal effect into an analysis. Prior beliefs reflect the analyst’s degree-of-uncertainty about a causal effect, and the Bayesian framework is the only paradigm of statistics that deals directly with this type of uncertainty.^1^

Finally, the administration and implementation of LSAs is, arguably, much more difficult than conducting a relatively small-scale randomized experiment. In addition to the sheer magnitude of the project, LSAs are often guided by political considerations that must be acknowledged and somehow addressed in the assessment design. Thus, this paper will also argue for some necessary conditions when implementing an LSA when the goal is to address a set of causal questions. These conditions have implications for the design of the assessments, and these implications will also be addressed in this paper. The present paper follows closely and synthesizes the work of Kaplan and his colleagues—in particular Kaplan (2009, 2014); Kaplan and Chen (2012); Chen and Kaplan (2015).^2^ The organization of this paper is as follows. I begin by providing an overview of the potential outcomes theory of Rubin (1974) as a framework for causal inference that I argue is appropriate with LSAs. I then outline the importance of the Bayesian perspective as a means of capturing uncertainty in all aspects of the causal inferential process. This is followed by a discussion of propensity score analysis which can be framed from both a classical (frequentist) perspective as well as a Bayesian perspective. Next, I argue for a series of necessary steps when addressing causal questions with LSAs. The paper closes with a discussion of the implications of addressing causal questions with large-scale educational assessments.

## The Rubin causal model: notation and definitions

An important set of papers that have provided the statistical foundations for causal inference in experimental and quasi-experimental studies derives from the work of Neyman (1923) and Rubin (1974), see also; Holland 1986) here referred to as the Rubin Causal Model (RCM). Their papers provide a framework for how statistical models that test causal claims are different from those that test associational claims, and that statistical theory has a great deal to add to the discussion of causal inference. Moreover, their work has led both the statistics and social science community to a deeper appreciation of the counterfactual theory of causation and has served as an impetus for extensions of the counterfactual theory of causation to the estimation of treatment effects (see e.g. Morgan and Winship 2007).

In outlining the RCM it is important to note that the terminology of “cause” is not confined to cases of randomized experiments. The notion of “cause” (or, interchangeably treatment) in the RCM is relative to some other cause. Specifically, in considering the phrase “attendance in full-day kindergarten causes higher scores in reading proficiency”, the idea is that attendance in full-day kindergarten causes *higher reading proficiency* relative to another cause—including the possibility of *“not attending kindergarten”*, or in our case “attending part-day kindergarten”. Holland (1986) states that “For causal inference, it is critical that each unit must be potentially exposable to any one of its causes”.

As an example, I might hypothesize that attending full-day kindergarten increases a student’s reading proficiency because I can also envision a student not attending full-day kindergarten but rather attending part-day kindergarten. That is, I can set up a sensible counterfactual conditional statement of the sort “what if the student was not exposed to full-day kindergarten”. Rubin (1974) thus links exposability to counterfactual propositions and the idea of a hypothetical experiment.

To formalize these ideas, Holland (1986) starts by defining a selection variable *S* that assigns a unit *i* (e.g. a student) who is a member of population to either a treatment condition, *T* = 1 or a control condition, *T* = 0. In randomized experiments, *S* is created by the experimenter, but in observational studies such as LSAs, assignment to a treatment condition often occurs naturally. In the RCM, the critical characteristic is that the value *Si* for each individual, *i*, could potentially be different.

The role of the outcome variable *Y* in the RCM is also crucial to the framework. First, for the variable *Y* to measure the effect of the cause, *Y* must be measured (or presumed to occur) post-exposure—that is after exposure to the treatment. Then, the value of the post-exposure outcome variable must be a result of either the cause *T* = 1 or the cause *T* = 0 defined on a particular student. Therefore, the RCM conceives of the same student providing an outcome variable after being exposed to the treatment, *Y*1*i* and after being exposed to the control *Y*0*i*. The causal effect defined within the RCM framework is then the difference between *Y*1 and *Y*0 for student *i*. That is for individual *i*, the goal, ideally, would be to observe the individual under receipt of the treatment and under non-receipt of the treatment. This, then, defines the *potential outcomes* framework for causal inference and can be expressed formally as

1 $$Y_{i}=T_{i}Y_{1i}\;+(1-T_{i})Y_{0i},$$

where *Yi* is the observed outcome of interest for individual *i*, *Y*1*i* is the potential outcome for individual *i* when exposed to the treatment, and *Y*0*i* is the potential outcome for individual *i* when not exposed to the treatment. however, as Holland (1986) points out, the potential outcomes framework has a serious problem—namely, it is rarely possible to observe the values of *Y*0 and *Y*1 on the same individual *i*, and therefore rarely possible to observe the effects of *T* = 1 and *T* = 0. Holland refers to this as the *fundamental problem of causal inference*.

A statistical solution to the Fundamental Problem offered by Holland (1986) is to make use of the population to which individual *i* belongs. In this case, the *average treatment effect*, can be defined (relative to the control group) as the expected value of the difference between *Y*1 and *Y*0 over the units in the population—viz.

2 $$E(Y_{1\;}\;-\;Y_{0})\;=\;ATE,$$

where *ATE* is the average treatment effect, simplified as

3 $$ATE=E(Y_{1})-E(Y_{0})$$

To quote Holland (1986), “The important point is that the statistical solution replaces the impossible-to-observe causal effect of *T* on a specific unit with the possible-to-estimate average treatment effect of *T* over a population of units” (p. 947. Italics in original).

Much more can be said about the RCM, but what must be discussed is Holland’s notion of what constitutes a cause, as his views are central to the arguments made in this paper. Holland writes

“Put as bluntly and as contentiously as possible... I take the position that causes are only those things that could, in principle, be treatments in experiments. The qualification, “in principle” is important because practical, ethical, and other considerations might make some experiments infeasible, that is, limit us to contemplating hypothetical experiments.”

In the final analysis, four points are crucial to an understanding of the RCM framework. First, the goal should be to seek out the effect of causes and not necessarily the causes of effects. For Holland, seeking out the causes of effects is valuable, but because our knowledge of causes is provisional, it is more valuable for a theory of causation to examine effects of causes. Second, effects of causes are always relative to other causes— particularly, the control. For Holland, and Campbell and Stanley (1966) before him, experiments that do not have a control condition are not experiments. Third, not everything can be a cause, and specifically, attributes cannot be causes. For example, an attribute of an individual, such as gender or race cannot be a cause since the notion of *potential exposability* of the unit to all levels of the treatment is not possible without also changing many other aspects of the individual. I cannot conceive of a situation in which I wish to know what a reading proficiency score would be if a female child were male, because potential exposability is simply not possible. In the context of attributes, all that can be derived are associations, and although associations are important and suggestive of variables that might moderate causal effects, they cannot be causes in the sense of the RCM framework. In other words, for Rubin (1974) and Holland (1986), there can be *“no causation without manipulation”* (Holland 1986, p. 959). That is, the RCM requires that the treatment is something under the direct or hypothetical manipulation of an investigator.

## Assumptions of the Rubin causal model

The Rubin causal model rests on two very important assumptions of relevance to its application with large-scale assessments. The first assumption is referred to as *strong ignorability of treatment assignment*, also referred to as *no confounding* or *no hidden bias*. Formally, the assumption of strong ignorability states

4 $$\{Y_{0},Y_{1}\}⊥T|Z,$$

where *Z* is a set of observed covariates. In words, Eq. (4) states that given a set of covariates *Z* the potential outcome under the treatment *Y*1 or control *Y*0 are independent of the treatment assignment mechanism. Strong ignorability will hold in randomized experiments where the treatment assignment (for a binary treatment) is obtained as a Bernoulli random variable. however, for observational data, the extent to which strong ignorability holds is dependent on *Z*.

The strong ignorability assumption is not plausible in LSAs or observational studies in general. This is because it is virtually impossible in observational studies to measure all necessary covariates that can be used to control for the non-random assignment of students to treatments. Any unobserved covariates that relate to treatment assignment will result in a violation of the strong ignorability assumption. Below I will consider the concept of the *causal field* discussed in Mackie (1974) to help restrict our measurement of covariates to those that are of immediate concern to the causal question at hand.

The second assumption underlying the Rubin Causal Model is the so-called *Stable Unit Treatment Value Assumption–SUTVA*. The SUTVA has two conditions. The first condition states that the treatment status of any unit does not affect the potential outcomes of the other units. Given that a treatment in the context of LSAs represents a self-reported (or parent-reported status (e.g. attendance in full-day or part-day kindergarten), it is unlikely that this part of SUTVA would be violated. The second condition of SUTVA is that the treatment for all units is comparable. This assumption is much harder to verify, particularly in international LSAs, because of the possibility of cross-national differences in the meaning of a particular causal variable.

## The Bayesian perspective

In the previous section I overviewed the RCM framework of causal inference that I argue is applicable to LSAs. Because, as noted by Holland, statistics plays a crucial role in the causal inference enterprise, a statistical framework is required to move to the next step of estimating the causal effect. Estimation of the causal effect of interest can proceed from the classical (frequentist) perspective or from the Bayesian perspective. I adopt a Bayesian perspective insofar as the Bayesian inferential paradigm represents a coherent system whereby all forms of uncertainty can be addressed when considering a causal question with LSAs. A general treatment of Bayesian statistics with applications to LSAs can be found in Kaplan (2014).

To briefly overview the Bayesian paradigm, denote by *Y* an outcome variable such as a student’s score on the ECLS-K reading proficiency assessment. Next, denote by *θ* a parameter that is believed to characterize the probability model of interest. For example *θ* could be the effect of attending full-day kindergarten (i.e. the regression coefficient on the dummy variable “attended full-day kindergarten or part-day kindergarten. Our concern is with determining the probability of observing *Y* given the unknown parameters *θ*, which I write as *p*(*Y*|*θ*). In statistical inference, the goal is to obtain estimates of the unknown parameters given the data.

The key difference between Bayesian statistical inference and frequentist statistical inference concerns the nature of the unknown parameters *θ*. In the frequentist tradition, the assumption is that *θ* is unknown but has a fixed value that we wish to estimate. In Bayesian statistical inference, *θ* is also considered unknown but instead is vieId as a random variable which needs to be described by a probability distribution that reflects our uncertainty about the true value of *θ*. Because both the observed data *Y* and the parameters *θ* are treated as random variables, we can model the joint probability of the parameters and the data as a function of the conditional distribution of the data given the parameters and the prior distribution of the parameters. More formally,

5 p(θ,Y)=p(Y|θ)p(θ),

where *p*(*θ*, *Y* ) is the joint distribution of the parameters and the data. Using Bayes’ theorem, we obtain the following

6 $$p(\theta \text{|}Y)\;=\;\frac{p(\theta ,\;Y)}{p(Y)}\;=\;\frac{p(Y\text{|}\theta \text{)}p(\theta \text{)}}{p(Y)}\;,$$

Note that the denominator of Eq. (6) does not involve model parameters, so we can omit the term and obtain the *unnormalized posterior distribution*

7 p(θ|Y)∝p(Y|θ)p(θ).

Equation (7) represents the core of Bayesian statistical inference and is what separates Bayesian statistics from frequentist statistics. In the context of our kindergarten-type attendance example, Eq. (7) states that our uncertainty regarding the effect of full-day kindergarten attendance on reading proficiency as expressed by the prior distribution *p*(*θ*), is *weighted* by the actual data *p*(*Y* |*θ*), yielding an updated estimate of our uncertainty, as expressed in the posterior density *p*(*θ*|*Y* ).

The immediate question that arises is how do we characterize our uncertainty about the effect of full-day kindergarten program attendance on reading proficiency? This is referred to as the “elicitation problem”, which has been discussed in detail in O’Hagan et al. (2006), and is beyond the scope of this paper. However, following the discussion given in Kaplan (2014), the general approach to specifying a prior distribution for the causal effect is to consider first what is reasonable to believe about the effect and to further consider the source of our belief. This issue has also been discussed by Leamer (1983) who orders priors on the basis of degree of confidence. Leamer’s hierarchy of confidence is as follow: truths (e.g. axioms) > facts (data) > opinions (e.g. expert judgement) > conventions (e.g. pre-set alpha levels). An interesting feature of this hierarchy, as noted by Leamer, concerns the inherent lack of “objectivity” in such choices as pre-set alpha levels, or any of a number of conventions used in frequentist statistics. Leamer (1983) goes on to argue that the problem should be to articulate exactly where a given investigation is located on this hierarchy. The strength of Bayesian inference lies precisely in its ability to incorporate existing knowledge into statistical specifications.

In the next section I discuss one method of causal inference—*propensity score analysis* that is directly situated within the RCM framework and has recently been extended to the Bayesian framework.

## Propensity score analysis

An implication of the RCM is that because we are unable to observe the outcomes of an individual under both treatment and control we need to find individuals in both groups that serve as each others’ counterfactuals. Thus, in order to warrant causal inferences in the setting of LSAs, individuals in treatment conditions should be matched as closely as possible to those in the control condition on observed pre-treatment assignment variables.

As a motivating example, consider again the effect of full- vs part-day kindergarten attendance on reading proficiency using data from ECLS-K (National Center for Education Statistics 2001). To warrant the claim that full-day kindergarten attendance increases reading proficiency, a researcher would need to find children who attended full-day kindergarten who are as similar as possible to those children who attended partday kindergarten on characteristics that might lead to selection into one or the other kindergarten program. These characteristics should have been measured (or hypothetically present) before the child’s selection into kindergarten program type (e.g. parental socio-economic status). Various forms of pre-treatment equating are available (see e.g. Rässler 2002; Rubin 2006). For this paper, I focus our attention on propensity score analysis as a method for equating groups on the basis of pre-treatment variables that are putatively related to the probability of having been observed in one or the other of the treatment conditions.

## The propensity score

In their seminal paper, Rosenbaum and Rubin (1983) proposed propensity score analysis as a practical tool for reducing selection bias through balancing treatment and control groups on measured covariates. Since then, a variety of propensity score techniques have been developed for both the estimation and the application of the propensity score. Models for estimating the propensity score equation have included parametric logit regression with chosen interaction and polynomial terms (e.g., Dehejia and Wahba 1999; Hirano and Imbens 2001a), and generalized boosting modeling (McCaffrey et al. 2004), to name a few. Methods for estimating the treatment effect while accounting for the propensity score include stratification, weighting, matching, and regression adjustment Guo and Fraser (2010).

More formally, consider first the potential outcomes model in Eq. (1). Under this model, the probability that individual *i* receives the treatment can be expressed as

8 $$e_{i}\;=p(T=1|Y_{1i},Y_{0i},Z_{i},U_{i}),$$

where *Ui* contain unobserved covariates. Notice that in an LSA, (*Y*0*i*, *Y*1*i*, *Ui*) are not observed. Thus, it is not possible to obtain the true propensity score. Instead, we estimate the propensity score based on covariates *Z*. Specifically,

9 ê(Z)=p(T=1|Z),

which is referred to as the *estimated propensity score*.

The estimated propensity score *e*ˆ(*Z*) has many important properties. Perhaps the most important property is the *balancing* property, which states that those in *T* = 1 and *T* = 0 with the same *e*ˆ(*Z*) will have the same distribution on the covariates *Z*. Formally, the balancing property can be expressed as

10 p{Z|T=1,ê(Z)}=p{Z|T=0,ê(Z)},

or equivalently as

11 T⊥Z|ê(Z).

## Implementation of the propensity score

There are four approaches that are commonly used in implementing the propensity score (a) stratification on ê(*Z*), (b) propensity score weighting, (c) optimal full matching, and (d) propensity score regression. Propensity score stratification involves forming strata directly on the basis of the observed propensity score. Subclassification into five strata on continuous distributions such as the propensity score has been shown to remove approximately 90 % of the bias due to non-random selection effects (Cochran 1968, see also Rosenbaum and Rubin 1983). However, for stratification on the propensity score to achieve the desired effect, the assumption of no hidden biases must hold.

Assuming no hidden biases, Rosenbaum and Rubin (1983) proved that when units within strata are homogeneous with respect to ê(*Z*), then the treatment and control units in the same stratum will have the same distribution on *Z*. Moreover, Rosenbaum and Rubin showed that instead of using all of the covariates in *Z*, a certain degree of parsimony can be achieved by using the coarser propensity score *e*ê(*Z*). Finally, Rosenbaum and Rubin (1983) showed that if there are no hidden biases, then units with the same value on a balancing score (e.g., the propensity score), but assigned to different treatments, will serve as each other’s control in that the expected difference in the responses of the units is equal to the average treatment effect.

Still another approach to implementing the propensity score is based on weighting. Specifically, propensity score weighting is based on the idea of Horvitz–Thompson sampling weights (Horvitz and Thompson, 1952), and is designed to weight the treatment and control group participants in terms of their propensity scores. Weights can be defined to yield either the average treatment effect or the average treatment effect on the treatment. The details of this approach can be found in Hirano and Imbens (2001b), Hirano et al. (2003), and Rosenbaum (1987).

The third common approach for implementing the propensity score is based on the idea of statistical matching (see e.g. Hansen 2004; Hansen and Klopfer 2006; Rässler 2002; Rosenbaum 1989). Following Rosenbaum (1989), consider the problem of matching a treated unit to a control unit on a vector of covariates. In observational studies, the number of control units typically exceeds the number of treated units. A *matched pair* is an ordered pair (*i*, *j*), with 1 ≤ *i* ≤ N and 1 ≤ *j* ≤ *M* denoting that the *i*th treated unit is matched with the *jth* control unit. As defined by Rosenbaum (1989), “*A complete matched pair* is a set Ј of *N* disjoint matched pairs, that is *N* matched pairs in which each treated unit appears once, and each control unit appears either once or not at all” (p. 1024).

Rosenbaum suggests two aspects of a “good” match. The first aspect is based on the notion of close matching in terms of a distance measure on the vector of covariates – for example, nearest neighbor matching. Obtaining close matches becomes more difficult as the number of covariates increases. Another aspect of a good match is based on covariate balance, for example, obtained on the propensity score. If distributions on the propensity score within matched samples are similar, then there is presumed to be balanced matching on the covariates.

Finally, the propensity score can be implemented directly into the regression that is used to estimate the treatment effect on the outcome. This is referred to as propensity score regression adjustment.

## Bayesian propensity score analysis

Propensity score analysis has been used in a variety of settings, including economics, education, epidemiology, psychology, and sociology. For comprehensive reviews see e.g. Guo and Fraser (2010), Steiner and Cook (2013), and Thoemmes and Kim (2011). Historically, propensity score analysis has been implemented within the frequentist perspective of statistics. In addition to the literature on frequentist-based propensity score analysis, there also exists literature examining propensity score analysis from a Bayesian perspective.

Rubin (1985) argued that because propensity scores are, in fact, randomization probabilities, a Bayesian approach to propensity score analysis should be of great interest to the applied Bayesian analyst, and yet propensity score estimation within the Bayesian framework was not addressed until relatively recently. Hoshino (2008) developed a quasi-Bayesian estimation method for general parametric models, such as latent variable models, and developed a Markov chain Monte Carlo (MCMC) algorithm to estimate the propensity score. McCandless et al. (2009) provided a practical Bayesian approach to propensity score stratification, estimating the propensity score and the treatment effect and sampling from the joint posterior distribution of model parameters via an MCMC algorithm. The marginal posterior probability of the treatment effect can then be obtained based on the joint posterior distribution. Similar to the McCandless et al. (2009) study, An (2010) presented a Bayesian approach that jointly models both the propensity score equation and outcome equation at the same time and extended this one-step Bayesian approach to propensity score regression and single nearest neighbor matching methods.

A consequence of the Bayesian joint modeling procedure utilized by McCandless et al. (2009) and An (2010) is that the posterior distribution of the propensity score may be affected by the outcome variable that are observed after treatment assignment, resulting in biased propensity score estimation. In order to maintain a fully Bayesian framework while overcoming the conceptual and practical difficulties of the joint modeling methods of McCandless et al. (2009) and An (2010), a two-step Bayesian propensity score approach (BPSA) was recently developed by Kaplan and Chen (2012) that can incorporate prior information on the model parameters of both the propensity score equation and outcome model equation. Consistent with Bayesian theory (see e.g., Kaplan 2014), specifying prior distributions on the model parameters is a natural way to quantify uncertainty—here in both the propensity score and outcome equations.

## A two‑step Bayesian propensity score analysis

A recent paper by Kaplan and Chen (2012) advanced a two-step approach to Bayesian propensity score analysis that was found to quite accurately estimate the treatment effect, while at the same time preventing undesirable feedback between the propensity score model and the outcome model.

In the Kaplan and Chen (2012) two-step Bayesian propensity score approach (hereafter, BPSA), the propensity score model specified was the following logit model.

12 $$Log\;\left(\frac{e(Z)}{1\;-\;e(Z)}\right)\;=\;\alpha \;+\;\beta Z,$$

where α is the intercept, β refers to the slope and *Z* represents a set of chosen covariates. For this step, Kaplan and Chen (2012) used the R package *MCMClogit*Martin et al. (2010) to sample from the posterior distributions of and β using a random walk Metropolis algorithm (Gilks et al., 1996). After the posterior propensity scores are obtained, a Bayesian outcome model is fit in the second step to estimate the treatment effect via various propensity score methods such as stratification, weighting and optimal full matching.

To illustrate their approach, Kaplan and Chen (2012) consider a posterior sampling procedure of a chosen Bayesian logit model with 1000 iterations and a thinning interval of 1. Then for each observation, there are *m* = 1000 posterior propensity scores *e*ˆ(*x*) calculated using propensity score model parameters and β as follows,

13 $$\text{ê}(x)\;=\;\frac{exp\;(\alpha \;+\;\beta x)}{1\;+\;exp(\alpha \;+\;\beta x)}.$$

Based on each posterior propensity score, there are J = 1000 posterior draws of the treatment effect generated from the posterior distribution of γ, where γ is the treatment effect. Assuming that *y* is the outcome and *T* is the treatment indicator, Kaplan and Chen (2012) then provide the following treatment effect estimator,

14 $$E(\gamma \;{|\;x,\;y,\;T)\;=\;m^{-1}\;J}^{-\;1}\;\sum_{i\;=\;1}^{m}\sum_{j\;=\;1}^{J}\gamma_{j}(\eta_{i}),$$

where $J^{-\;1}\;\sum_{j\;=\;1}^{J}\gamma_{j}\;(\eta_{i})$ is the posterior sample mean of γ in the Bayesian outcome model based on the ith set of propensity scores ηi, i = 1,...,*m* and *j* = 1,...,*J*. This posterior sample mean is then averaged over m sets of posterior propensity scores. The posterior variance of γ is then based on the total variance formula,

15 $$\text{Var (}\gamma {\text{ | x,y,T) = m}}^{-1}\;\sum_{i\;=\;1}^{m}\sigma_{\gamma (\eta_{i})}^{2}\;+\;{\left(m\;-\;1\right)}^{-\;1}\;\sum_{i\;=\;1}^{m}\left\{\mu_{\gamma \;(\eta_{i})}\;-\;m^{-1}\;\sum_{i\;=\;1}^{m}\mu_{\gamma (\eta_{i})}\right\}{\;}^{2},$$

Where

16 $$\sigma_{\gamma (\eta_{i})}^{2}\;=\;{\left(J\;-\;1\right)}^{-\;1}\;\sum_{j\;=\;1}^{J}\left[\left\{\gamma_{j}(\eta_{i})\;-\;J^{-\;1}\;\sum_{j\;=\;1}^{J}\gamma_{j}(\eta_{i})\right\}\right]{\;}^{2}\;,$$

is the posterior sample variance of γ in the Bayesian outcome model under the *i*th set of propensity scores and

17 $$\mu_{\gamma \;(\eta_{i})}\;=\;J^{-\;1}\;\sum_{j\;=\;1}^{J}\gamma_{j}(\eta_{i}),$$

is the posterior sample mean of γ in the same Bayesian outcome model. Notice that two sources of variation are present in Eq. (15). The first source of variation is the average of the posterior variances of *γ* across the posterior samples of propensity scores, represented by the first part of the right hand side of Eq. (15), and the second source of variation comes from the variance of the posterior means of γ obtained across the posterior samples of propensity scores, estimated by the second part of the right of hand side of Eq. (15) Kaplan and Chen (2012).

Kaplan and Chen (2012) conducted three simulation studies as well as a small case study comparing frequentist propensity score analysis with the two-step Bayesian alternative focusing on the estimated treatment effect and variance estimates. The effects of different sample sizes, true treatment effects and choice of priors on the treatment effect and variance estimates were also evaluated. Consistent with Bayesian theory, Kaplan and Chen’s (2012) findings showed that lower prior precision of the treatment effect is desirable when no prior information is available in order to obtain estimates similar to frequentist results but with wider intervals that properly capture the uncertainty in the treatment effect; or, higher prior precision is preferable when accurate prior information regarding treatment effects is attainable in order to obtain more precise treatment effect estimates and narrower intervals. For the case of small sample size, the Bayesian approach shows slight superiority in the estimation of the treatment effect compared to the frequentist counterpart.

The case study in Kaplan and Chen (2012) used data from the Early Childhood Longitudinal Study Kindergarten Cohort of 1998 (ECLS-K) National Center for Education Statistics (2001). The ECLS-K is a nationally representative longitudinal sample providing comprehensive information from children, parents, teachers and schools. The sampled children comes from both public and private schools and attends both full-day and part-day kindergarten programs, having diverse socioeconomic and racial/ethnic backgrounds.

In their case study, Kaplan and Chen examined the treatment effect of full versus part day kindergarten attendance on IRT-based reading scores for children at the end of 1998 fall kindergarten. A sample of 600 children was randomly selected proportional to the number of children in full or part day kindergarten in the population. This resulted in 320 children in full day kindergarten and 280 children in part day kindergarten. Thirteen covariates were chosen for the propensity score equation. These included gender, race, child’s learning style, self-control, social interactions, sadness/loneliness, impulsiveness/ overreactiveness, mother’s employment status, whether first time kindergartner in 1998, mother’s employment between birth and kindergarten, non-parental care arrangements, social economic status and number of grandparents who live close by. Missing data were handled via the R program *mice* (multivariate imputation by chained equations) (van Buuren and Groothuis-Oudshoorn, 2011). Non-informative uniform priors were used for both the propensity score equation and the outcome equation. The MCMC sampling required 400,000 iterations with burnin 5000 and thin interval 400, which significantly reduced autocorrelation to an acceptable range.

Compared to the nonsignificant results estimated by simple regression, both PSA and BPSA were able to detect the significant treatment effect and greatly reduced the estimation bias. The Bayesian approach with little prior information achieved similar estimated treatment effects compared to the conventional frequentist approach, but offered a better variance estimate, taking into account the uncertainty of propensity scores and therefore having wider credible intervals. On average, the Bayesian stratification method had 6.2 % wider interval than conventional approach, the Bayesian weighting approach achieved an 8.9 % wider interval, and the Bayesian optimal full matching method obtained as much as 14 % wider interval. This result agreed with McCandless et al. (2009) and was consistent with Kaplan and Chen’s (2012) simulation results and Bayesian theory.

A further study of the covariate balance properties of the Kaplan and Chen (2012) approach was given in a case study by Chen and Kaplan (2015). Their results revealed that both Bayesian and frequentist propensity score approaches substantially reduced initial imbalance as expected, and their performance on covariate balance was similar in regard to the standardized mean/proportion differences and variance ratios in the treatment group and control group. Similar performance was also found with respect to the 95 % bootstrap intervals and posterior probability intervals. That is, although the frequentist propensity score approach provided slightly better covariate balance for the propensity score stratification and weighting methods, the two-step Bayesian approach offered slightly better covariate balance under optimal full matching method. Results of the Chen and Kaplan (2015) simulation study indicated similar findings. In addition, the Bayesian propensity score approach with informative priors showed equivalent balance performance compared to the Bayesian approach with non-informative priors, indicating that the specification of the prior distribution did not greatly influence the balance properties of the two-step Bayesian approach. The optimal full matching method, on average, offered the best covariate balance compared to stratification and weighting methods for both Bayesian and frequentist propensity score approaches. Chen and Kaplan (2015) also found that the two-step Bayesian approach under optimal full matching with highly informative priors provided, on average, the smallest standardized mean/proportion difference and variance ratio of the covariates between the treatment and control groups.

Chen and Kaplan (2015) argued that a benefit of conducting Bayesian propensity score analysis is that one can obtain the posterior distribution of the propensity score and thus the posterior distribution of corresponding balance indices (e.g. Cohen’s *d* and variance ratio) so that the variation in balance indices can be studied in addition to the point estimates to assist in balance checking. Good balance is achieved if both the point estimates and the posterior probability intervals of the balance indices fall into the desirable range.

## Bayesian model averaging for PSA

The distinctive feature that separates Bayesian statistical inference from its frequentist counterpart is its focus on describing and modeling all forms of uncertainty. The primary focus of uncertainty within Bayesian inference concerns prior knowledge about model parameters. however, within the Bayesian framework, parameters are not the only unknown elements. In fact, the Bayesian framework recognizes that model choice possess uncertainty insofar as a particular model is typically chosen based on prior knowledge of the problem at hand and the variables that have been used in previously specified models. This form of uncertainty often goes unnoticed. his problem was succinctly stated by Hoeting et al. (1999) who write

“Standard statistical practice ignores model uncertainty. Data analysts typically select a model from some class of models and then proceed as if the selected model had generated the data. This approach ignores the uncertainty in model selection, leading to over-confident inferences and decisions that are more risky than one thinks they are.” (p. 382)

An internally consistent Bayesian framework for model building and estimation must also account for model uncertainty. The current approach to addressing the problem of uncertainty lies in the method of *Bayesian model averaging* (BMA) (Hoeting et al. (1999, 1996); Madigan and Raftery 1994).

In outlining BMA consider a quantity of interest such as a future observation or a parameter. Following Madigan and Raftery (1994), I denote this quantity as .Δ. Next, consider a set of competing models *Mk, k = 1, 2,...,K* that are not necessarily nested. The posterior distribution of . Δ. given data *D* can be written as

18 $$p(\Delta \text{|D)}\;\text{=}\;\sum_{k\;=\;1}^{K}p(\Delta \text{|}M_{k})p(M_{k}\text{|}D),$$

where p(Mk|D) is the posterior probability of model Mk written as

19 $$p(M_{k}\text{|}D)\;=\;\frac{p(D\text{|}M_{k})p(M_{k})}{\sum_{l\;=\;1}^{K}p(D\text{|}M_{l})p(M_{l})},\;\;\;\;\;\;\;l\;\neq \;k.$$

The interesting feature of Eq. (19) is that p(Mk|D) will likely be different for different models. The term p(D|Mk) can be expressed as an integrated likelihood

20 $$p(D\text{|}M_{k})\;=\;\int p(D\text{|}\theta_{k},\;M_{k})p(\theta_{k}{\text{|M}}_{k})d\theta_{k},$$

where *p*(*θk* |*Mk* ) is the prior density of *θ*k under model *Mk* Raftery et al. (1997). Thus, Bayesian model averaging provides an approach for combining models specified by researchers, or perhaps elicited by key stakeholders.

As pointed out by Hoeting et al. (1999), Bayesian model averaging is difficult to implement. In particular, they note that the number of terms in Eq. (18) can be quite large, the corresponding integrals are hard to compute, and choosing the class of models to average over is also challenging. To address the problem of computing Eq. (20) the Laplace method can be used and this will lead to a simple BIC approximation under certain circumstances (Tierney and Kadane 1986; cited in Hoeting et al. 1999). Th problem of reducing the overall number of models that one could incorporate in the summation of Eq. (18) has led to two interesting solutions. One solution is based on the so-called *Occam’s window* Madigan and Raftery (1994) and the other is based on *Markov chain Monte Carlo Model composition* (MC^3^) A discussion of the algorithms is beyond the scope of this paper. Suffice to say that the advantage of BMA has been discussed in Madigan and Raftery (1994) who showed that Bayesian model averaging provides better predictive performance than that of a single model based on the log score rule Hoeting et al. (1999).

In a recent paper Kaplan and Chen (2014), investigated the use of Bayesian model averaging in propensity score analysis in a simulation study and a case study again using data from ECLS-K. Kaplan and Chen (2014) approximated Bayesian model averaging approach based on the model-averaged propensity score estimates produced by the R package *BMA*, but which ignored uncertainty in the propensity score itself. Therefore, Kaplan and Chen (2014) provided a fully Bayesian model averaging approach via MCMC to account for uncertainty in both parameters and models. A detailed study of their approach examined the differences in the causal estimate when incorporating noninformative versus informative priors in the model averaging stage. Kaplan and Chen (2014) also assessed the predictive performance of both Bayesian model averaging propensity score approaches and compare it to the case without Bayesian model averaging. Overall, their results showed that both Bayesian model averaging propensity score approaches recovered the treatment effect estimates well and generally provide larger uncertainty estimates, as expected. Both Bayesian model averaging approaches offered slightly better prediction of the propensity score compared to the Bayesian approach with a single propensity score equation. Covariate balance checks for the case study showed that both Bayesian model averaging approaches offered good balance. The fully Bayesian model averaging approach also provided posterior probability intervals of the balance indices

## Necessary conditions for causal inference in LSAs

The view regarding the appropriateness of drawing causal inferences with LSAs advocated in this paper rests on several necessary conditions. I view these conditions as essential regardless of whether one adopts a Bayesian approach to causal inference or situate our investigation in the frequentist framework. However, as noted earlier, I view the Bayesian framework as more flexible insofar as it can account for all the layers of uncertainty in statistical models for causal inference. The necessary steps are as follows and more fully developed below in the context of our kindergarten program type example.

1. A well defined causal question stemming from a theoretical framework that is presumably of interest to governing bodies responsible for policy priorities.
2. A causal question framed as a counterfactual question capable of yielding a real-life manipulation or intervention within the framework of a randomized experiment.
3. The collection of ancillary covariate information relevant to the causal question of interest.
4. The choice of a statistical method that provides an appropriate causal estimand accounting for the ancillary covariate information and a sequence of sensitivity analyses that examine changes in the causal estimand across a range of plausible confounding relationships.

### Condition 1: a well‑defined causal question

The administrative structure of LSAs usually contains an overarching governing board representing key stakeholders. For example, PISA is governed by the *PISA Governing Board* which is made up of representatives of the PISA participating countries and who set the policy priorities for PISA. These policy priorities become instantiated through the various frameworks produced by the contractors chosen to implement PISA, with input from outside expert groups and mediated by the OECD Secretariat. A similar structure exists for large-scale surveys administered by the IEA. Thus, central to the use of LSAs for causal inference is the articulation of one or more priority causal questions agreed upon by the governing body and further articulated through the frameworks. From our example, a governing body might agree that the issue of attendance in full-day kindergarten programs is important in terms of its purported linkage to reading proficiency. This policy priority would be communicated to the framework developers and eventually to those charged with writing the questionnaire items.

### Condition 2: counterfactual propositions

Given a well-defined causal question that is of policy priority, the next step is to articulate the question in the form of a counterfactual conditional statement. Recall that a counterfactual conditional statement is a subjunctive phrase in of the form “if *T* had not occurred, *Y* would not have occurred”. This form of causal reasoning is intimately connected to the RCM insofar as the RCM presumes that a unit of observation could have two potential outcomes under different conditions of a treatment *T*, including “not *T*”. In this section I review the work of Mackie (1974), as it is his work on counterfactual propositions that I argue is of most value to causal inference with LSAs. The specific form of the question must have cross-cultural comparability when interest is in comparative causal inference with international LSAs. Developing a well articulated counterfactual proposition is a crucial component of the necessary conditions for causal inference with LSAs and so I discuss this issue at length next. For an additional detailed study of counterfactuals from the philosophical tradition, see Lewis (1973). An excellent review of counterfactuals within social science research can be found in Morgan and Winship (2007).

#### Mackie and the INUS condition

In this section, I outline Mackie’s important contribution to our understanding of causation, as developed in his seminal work *The Cement of the Universe* (1974). I concentrate on two specific aspects of Mackie’s work on causation because his ideas lay a strong logical groundwork for how to consider causal inference in LSAs. The first aspect of Mackie’s work addresses a regularity theory of causation and the second aspect concerns a conditional analysis of causation. It should be understood that Mackie’s overall contributions are much deeper than I have the space to present.

To begin, Mackie (1974) situates the issue of causation in the context of a modified form of the counterfactual conditional statement if X causes Y, then this means that X occurred and Y occurred, and Y would not have occurred if X had not. This strict counterfactual statement is problematic for the following reason; I can conceive of *Y* occurring if *X* had not. For example, I can conceive of improved reading proficiency without exposure to early literacy programs. Thus, if I am to attribute improved reading proficiency to exposure to early literacy programming, I must define the conditions under which the exposure took place.

Mackie suggests that the problem in distinguishing between conditions and causes is addressed by considering that causes take place in a context, or what Mackie refers to as a *causal field*. For Mackie

“Both cause and effect are seen as differences within a field; anything that is not part of the assumed (but commonly understated) description of the field itself will, then, be automatically ruled out as a candidate for the role of cause”.

Mackie goes on to say

“What is said to be caused, then, is not just an event, but an event-in-a-certainfield, and some ‘conditions’ can be set aside as not causing this-event-in-this-field simply because they are part of the chosen field, though if a different field were chosen, in other words if a different causal question were being asked, one of those conditions might well be said to cause this-event-in-that-other-field.” (p. 35)

In the context of a causal field, there can be a host of *factors* that could qualify as causes of an event. Following Mackie (1974) let *A, B, C..., etc*, be a list of factors that lead to some effect whenever some conjunction of the factors occurs. A conjunction of events may be *ABC* or *DEF* or *JKL*, etc. This allows for the possibility that *ABC* might be a cause or *DEF* might be a cause, etc. So, all (*ABC* or *DEF* or *JKL*) are followed by the effect. For simplicity, assume the collection of factors is finite, that is only *ABC*, *DEF*, and *JKL*. Now, this set of factors (*ABC* or *DEF* or *JKL*) is a condition that is both necessary and sufficient for the effect to occur. Each specific conjunction, such as *ABC* is sufficient but not necessary for the effect. In fact, following Mackie, *ABC* is a “minimal sufficient” condition insofar as none of its constituent parts are redundant. That is, *AB* is not sufficient for the effect, and *A* itself is neither a necessary nor sufficient condition for the effect. however, Mackie states that the single factor, in this case, *A*, is related to the effect in an important fashion—viz. “[I]t is an *insufficient* but *non-redundant* part of an *unnecessary* but *sufficient* condition: it will be convenient to call this ... an *inus* condition.” (p. 62)

It may be useful to examine Mackie’s ideas in the context of our full-day kindergarten program attendance and reading proficiency example. Mackie’s concept of *inus* conditions alerts us to the importance of carefully specifying the causal field in which causal claims regarding the full-day kindergarten program attendance are made, and to attempt to isolate those factors that serve as *inus* conditions for causal inferences. Specifically, in the context of examining policies or interventions centered on improving reading proficiency in young children, Mackie would have us first specify the causal field or context under which the development of reading proficiency. I could envision a large number of factors that could qualify as causes of reading proficiency. In Mackie’s analysis, the important step would be to isolate the set of conjunctions, any one of which might be necessary and sufficient for improved reading proficiency. A specific conjunction might be attendance in pre-primary education, parental support and reading involvement, teacher training. This set is the minimal sufficient condition for reading proficiency in that none of the constituent parts are redundant. Any two of these three factors is not sufficient for reading proficiency, and one alone—say focusing on pre-primary education, is neither necessary nor sufficient. however, full-day kindergarten program attendance is an *inus* condition for reading proficiency. That is, the emphasis on full-day kindergarten program attendance is insufficient as it stands, but it is also a non-redundant part of a set of unnecessary but (minimally) sufficient conditions.

#### Woodward and the manipulability theory of causation

Mackie’s notions of causal fields and the *inus* condition are essential in providing a deeper background for a counterfactual theory of causation. however, Mackie does not provide specific advice with regard to developing notions of causal explanation. More recently, a *manipulability theory* of causation was put forth by Woodward (2003) as an attempt to provide a foundation for causal explanation. For Woodward (2003), a causal explanation is an explanation that provides information for purposes of manipulation and control. To quote Woodward

“... my idea is that one ought to be able to associate with any successful explanation a hypothetical or counterfactual experiment that shows us that and how manipulation of the factors mentioned in the explanation ... would be a way of manipulating or altering the phenomenon explained...Put in still another way, an explanation ought to be such that it can be used to answer what I call the what-if-things-hadbeen-different question...” (p. 11)

It is certainly the case that the experimental approach allows one to ask the *what-ifthings-had-been-different* question. Note that Woodward’s reasoning is the centerpiece of the RCM framework because it bases this question at the level of the individual.

At the forefront of Woodward’s manipulability account of causal explanation is the idea of a hypothetical experiment. however, Woodward makes clear that experiments are not the only way that one can learn about causal relationships. Under certain assumptions, one can learn about causal relationships from a combination of observation and experiment. Woodward writes

“A plausible manipulability theory will not deny that reliable causal inference on the basis of non-experimental evidence is possible, but rather, suggests a specific way of thinking about such inferences: I should think of them as an attempt to determine (on the basis of other kinds of evidence) what the results of a suitably designed hypothetical experiment of manipulation would be without actually carrying out this experiment.”

I argue that Mackie’s theories of causal fields and *inus* conditions provide a philosophical foundation for Woodward’s manipulability theory of causal explanation. Specifically, articulating the causal field and identifying an *inus* condition for causality is not enough. We need an account of how identifying an *inus* condition for causation provides a possible explanation for some observed effect. Woodward’s detailed account of manipulation and intervention, along with the crucial notion of invariance, provides, in our view, precisely the grounding needed to move forward to a non-experimental/observational approach to causal inference in the context of LSAs. however, what is required is a methodology for testing causal explanations with survey data that provides value-added by moving us beyond the relatively simple causal accounts gleaned from randomized experimental designs. In my view, Bayesian statistical methods framed within the Rubin Causal Model may provide such a methodology.

### Condition 3: collecting ancillary covariates

A clear implication of Mackie’s notion of a causal field and Woodward’s manipulability account of causation for causal inference in LSAs is the need to collect as many relevant ancillary covariates as possible. Mackie’s notions of a causal field and the resulting *inus* condition for causal inference is helpful in narrowing down the number of covariates to be collected; however there still remains a number of practical concerns. First and foremost is the collection of the “right” covariates. The concept of the causal field notwithstanding, it still remains that relevant covariates need to be chosen and measured to help insure that strong ignorability holds given the observed covariates. Naturally this falls in the domain of the content experts who advise contractors and governing bodies as to the relevant covariates to be collected in support of the priority causal questions. For example, guided by policy priorities, experts in early childhood education and in reading would work to develop a list of possible covariates that could be used in a propensity score analysis for modeling the non-random selection into pre-primary education.

A practical problem that still remains, however, is the space in the survey necessary to add such questions insofar as questionnaires contain not only needed demographic information but also trend information across cycles of the survey. Possible solutions to this space problem involve questionnaire rotation design (see e.g. Kaplan and Su 2016; Gonzalez and Rutkowski 2010; Rutkowski 2011; von Davier 2013) or optional country questionnaires; these are areas for further research and development.

### Condition 4: choosing a statistical model

For this paper, I chose to discuss Bayesian propensity score analysis as one of many possible statistical models for estimating causal effects with LSAs. The issue is not so much what paradigm of statistics one identifies with (Bayesian or frequentist), but rather that the statistical model matches the causal question of interest and allows for assessing the sensitivity of the statistical model to violations of the assumptions underlying the causal modeling framework. Thus, in addition to propensity score analysis described in this paper, other methods such as *causal mediation analysis* Imai et al. (2010a, b, 2011) and its Bayesian extensions (Park and Kaplan, 2015) might be applicable to causal questions with LSAs. What matters is whether such methods yield the causal estimand of interest and whether the obtained estimand is capable of being evaluated against violations of causal assumptions.

The issue of assessing whether the causal estimand is sensitive to violations of causal assumptions concerns the sensitivity to hidden bias. Specifically, a sensitivity analysis allows the researcher to assess changes to the causal estimand based on incorporating a series of reasonable values for the parameters relating the unobserved confounders to the observed covariates and causal variable. Substantively important changes to the causal parameter due to small changes in the magnitude of the hidden bias can lead to bounds being placed on the causal estimand. A sensitivity analysis of this sort is consistent with the Bayesian statistical framework insofar as hidden bias parameters can be set as priors with specified precision reflecting prior knowledge about possible hidden biases.

#### Eliciting priors

Condition 4 mentioned above focused on choosing the correct statistical model for the causal question of interest. however, if one does adopt a Bayesian approach to causal inference, the question of priors comes to the forefront. To reiterate, perhaps the singular advantage of the Bayesian school of statistics is that it provides a way to elicit and directly incorporate prior knowledge into a study. The frequentist school treats each study as if it is the first of its kind, and that no prior information is available on the topic at hand. however, even a casual consideration of standard frequentist practice reveals that this is patently untrue. Perhaps the most obvious example within frequentist practice is the choice of variables to be included in a model. This choice is most certainly made on the basis of prior information; but given that there are likely alternative interpretations of that prior information, the uncertainty in the choice is not made explicit. The Bayesian school, in stark contrast, incorporates prior knowledge into an analysis that is open to scrutiny by the scientific community and provides an immediate assessment of the analyst’s view of the degree of uncertainty entering into his/her parameters and models.

#### Subjective Bayes

In a similar vein, the kind of prior knowledge that can be entered into a Bayesian framework can be “subjective” or “objective”. Subjective Bayesian practice attempts to bring prior knowledge about what is reasonable to believe about a parameter directly into an analysis. This prior knowledge represents the analysts (or others) degree-of-belief, which I prefer to consider as one’s “degree-of-uncertainty”. An analyst’s degree-of-uncertainty is encoded directly into the specification of the prior distribution, and in particular on the degree of precision around the parameters of interest.

Press (2003) notes that there are advantages and disadvantages to adopting subjective Bayesian practice which I summarize here. Of relevance to the the use of Bayesian methods for causal inference, the major advantage in using subjective priors is that it is the only way to encode prior research findings into an analysis. In the context of LSAs, relatively “objective” prior knowledge can come from prior administrations of the assessment. However, encoding even objective prior knowledge into the prior distribution can be difficult. Moreover, subjective priors may not always be appropriate for public policy situations because other researchers as well as policy stakeholders may hold different priors.^3^

#### Objective Bayes

What if objective data from prior LSA administrations is unavailable? In the absence of prior data and perhaps the inability to obtain expert opinion, then so-called “objective” priors can be implemented. The advantages of objective priors, as pointed out by Press (2003) is that (a) objective priors can be used as benchmarks against which choices of other priors can be compared, (b) objective priors reflect the view that little information is available about the process that generated the data, (c) there are cases in which the results of a Bayesian analysis with an objective prior provides results equivalent to those based on a frequentist analysis, and (d) objective priors are sensible public policy priors insofar as they allow for policy analysis without incorporating the prior knowledge of the analyst.

A major problem with objective priors is that they are hard to defend—particularly in the context of research with LSAs. For example, perhaps the most extreme version of an objective prior is the so-called *uniform prior* which encodes total ignorance about the average value and precision of a parameter. For example, in the case of pre-primary education, using a uniform prior distribution ranging from −∞to +∞ would say that all values of the causal effect across the real numbers are equally likely. It should be noted, however, that the Bayesian literature has developed a large number of objective (aka “reference”) priors that can also be used for Bayesian inference as well as comparisons to models that use subjective priors (e.g, Jeffreys’ prior or the maximum entropy prior); but these priors simply provide different ways of quantifying the notion of complete uncertainty about a causal effect. Nevertheless, I agree with Berger (2006), that reference priors should be used “in scenarios in which a subjective analysis is not tenable”, although I believe that these scenarios are now rare in the world of LSA.

#### An aside: handling multilevel data

Our discussion so far has focused on causal inference in LSAs where the policy question of interest concerned student reported attendance in full or part-day kindergarten. Large-scale assessment data, however, are typically generated from a clustered sampling design where (within countries) schools are sampled first followed by sampling students within schools. It is well known that statistical modeling of any sort that ignores the clustered nature of the data can lead to biased estimates regardless of whether the estimates have a causal interpretation or simply represent associations. In the context of causal inference, however, it is important to note that selection into treatment conditions may vary across schools due to the specific features of local policy. Thus, particularly for propensity score analysis, it is necessary to have a method to address differential selection mechanisms across schools.

Recent work by Kim and Steiner (2015) provide an approach for addressing differential selection across schools through the use of latent class models for across-school matching. The essential idea is to identify clusters of schools that are similar with respect to the propensity score model. This is accomplished by specifying a multilevel latent class logit model (see e.g. McLachlan and Peel (2000) that yields the probability that, say, student i (i = 1, 2,...n) in school g (g = 1, 2,...G) in latent class c (c = 1, 2,...,C) selected to receive the treatment. This probability is modeled as a function of level–1 and level–2 covariates. By adding a latent class component to the multilevel logit model, Kim and Steiner (2015) are able to identify classes of schools that share similar selection mechanisms but also different causal estimands for different latent selection classes of schools. The approach advanced by Kim and Steiner (2015) provides a nuanced assessment of the treatment effect of interest while at the same time accounting for the multilevel nature of the data.

The approach developed by Kim and Steiner (2015) is situated within frequentist framework of statistics. Their approach could be implemented within a Bayesian framework by recognizing that the multilevel logit model can be specified as a Bayesian hierarchical model (Kaplan, 2014). First, as usual, priors would have to be assigned to all model parameters. However, in the case of Kim and Steiner (2015) approach which is based on latent class analysis, the latent selection classes are assumed to follow a multinomial distribution with parameters, say, π = (π_1_,π_2_,...,π_C_) representing latent class probabilities. The conjugate prior for is the Dirichlet (π_1_,π_2_,...,π_C_) prior (see e.g. Evans et al. (2000). For a discussion of Bayesian latent class analysis, see Gelman et al. (2013) and Kaplan (2014).

## Discussion

This paper provided a review and synthesis of the problem of causal inference in large-scale educational assessments from a Bayesian perspective. I proposed an approach to causal inference in LSAs that requires the articulation of framework for causal inference followed by a statistical approach that closely matches the framework and can yield the causal estimand of interest. For this paper, I situated causal inference with LSAs in the framework of the Rubin Causal Model Rubin (1974). The Rubin Causal Model rests on the the notion of potential outcomes, which, in turn requires us to consider causal variables as representing hypothetically manipulable policies or interventions. I next chose the Bayesian paradigm of statistical inference as the most coherent and natural approach to assessing causal effects within the Rubin Causal Model. My choice of the Bayesian paradigm rested on the view that all forms of uncertainty within the causal inferential enterprise should be made explicit, and that the Bayesian approach is uniquely suited to this end. Finally, I provided a set of conditions that I argued is necessary for conducting causal inference with LSAs.

The enterprise of LSAs is complex and multi-faceted; attempting to balance political/ policy priorities with the technical requirements necessary to yield reliable and valid data. In my view, the political/policy priorities need to be addressed first. That is, the governing bodies of LSAs must first decide if addressing the effects of specific causes is a policy priority, and then to focus on a small set of priority causal questions. Given the operational concerns mentioned earlier, it will not be easy to balance a priority focus on causal inference with the other real demands placed on LSAs. however, should there be an interest in addressing causal questions with LSAs, I argue that the framework and methodology developed in this paper serve as a starting point for engaging in causal inquiry with LSAs.

Of course, additional support for basic research on causal inference with large-scale assessments is needed. First, the methods described in this paper need to be developed more fully and tested on extant large-scale assessment data, and concurrently, new software must be developed to support the statistical models proposed in this paper. Second, it is important to study precisely how causal variables can be reliably measured and used in statistical models such as those described in this paper. This issue pertains to the second condition of SUTVA—that the treatment for all units is comparable—and this is especially true when interest is focused on comparative causal inference with international large-scale assessments. The field-trial stage of large-scale assessment operations might provide a fruitful testbed for this research. Moreover, it must be noted that my example was one of a simple binary treatment. Clearly, pre-primary education is a market basket of quite specific sets of activities, each of which could serve as treatments in their own right. Here again, this issue is less one of the framework of causal inference or the statistical method, but rather one of fruitful collaboration among content experts guided by policy priorities with testing and evaluation of causal variables within the field-trial stage. Finally, alternative frameworks for causal inference should be studied in terms of their value in the context of large-scale assessments. The hope is that this paper stimulates a broader discussion of the challenges and opportunities of causal inference with large-scale assessments.

## Footnotes

1 In the frequentist domain, the standard error of a parameter captures the sampling variability of the parameter under hypothetical repeating sampling from a population; it does not capture uncertain knowledge about the parameter itself.

2 Permission to draw on this material has been obtained from Guilford Press, Springer Publications and Taylor and Francis. Proof of permission is available upon request.

3 However, models with different priors can be compared in terms of their predictive accuracy using such methods as *Bayes factors*.
